# HER3 and FOLR1 Expression as Actionable Targets in High-Grade Serous Ovarian Carcinoma: Prognostic and Therapeutic Implications

**DOI:** 10.3390/medicina62030492

**Published:** 2026-03-05

**Authors:** Nurhan Onal Kalkan, Ramazan Oguz Yuceer, Seyhmus Kaya, Nurgul Dogru, Ayhan Yıldırım

**Affiliations:** 1 Department of Clinical Oncology, Batman Training and Research Hospital, 72070 Batman, Turkey; 2 Department of Pathology, Cumhuriyet University School of Medicine, 58140 Sivas, Turkey; r.yuceer66@hotmail.com (R.O.Y.); drseyhmuskaya21@gmail.com (S.K.); 3 Department of Pathology, Batman Training and Research Hospital, 72070 Batman, Turkey; nurguldogru@hotmail.com; 4 Department of Pathology, Gazi Yaşargil Training and Research Hospital, 21010 Diyarbakır, Turkey; drayhanyildirim@gmail.com

**Keywords:** high-grade serous ovarian carcinoma, HER3, FOLR1, prognosis, immunohistochemistry, antibody–drug conjugates, biomarkers

## Abstract

*Background and Objectives*: High-grade serous ovarian carcinoma (HGSC) is characterized by aggressive tumor behavior, frequent recurrence, and limited long-term survival. Despite the established clinicopathological prognostic factors, significant heterogeneity in clinical outcomes persists, highlighting the need for biologically relevant molecular biomarkers. HER3 and folate receptor alpha (FOLR1) have promising prognostic biomarkers in ovarian cancer; however, the combined biological and prognostic impact of these two molecules has not yet been clearly demonstrated. *Materials and Methods*: This retrospective observational study included 66 patients with histopathologically confirmed HGSC. The immunohistochemical expression of HER3 and FOLR1 was evaluated using a standardized immunoreactivity scoring system. Associations with clinicopathological features were analyzed, and survival outcomes were analyzed using Kaplan–Meier analysis and Cox proportional hazards regression models. *Results*: High HER3 expression was significantly associated with distant metastasis and was identified as an independent adverse prognostic factor for both overall survival (OS) and progression-free survival (PFS). FOLR1 expression was associated with OS in univariate analysis, but did not retain independent prognostic significance in multivariate models. A moderate yet statistically significant positive correlation between HER3 and FOLR1 expression was observed, suggesting a potential association between proliferative signaling and metabolic pathways that may warrant further mechanistic investigation. *Conclusions*: Our findings demonstrate that HER3 is a robust prognostic biomarker in HGSC and support a biologically relevant HER3–FOLR1 interaction contributing to tumor aggressiveness. These results provide a translational rationale for combined biomarker assessment and for the development of HER3- and FOLR1-targeted therapeutic strategies, particularly antibody–drug conjugates, for HGSC.

## 1. Introduction

Ovarian cancer is a gynecological malignancy with the highest mortality rate among women, ranking approximately eighth in incidence and fifth in cancer-related deaths worldwide [1]. High-grade serous ovarian carcinoma (HGSC), which accounts for nearly 70% of all ovarian cancers, is characterized by aggressive biological behavior, early tendency for peritoneal dissemination, and high recurrence rates [2].

The current standard treatment approach consists of optimal cytoreductive surgery, followed by platinum-based chemotherapy. Despite an initial clinical response, disease progression and mortality occur in a substantial proportion of patients [3]. Although HGSC is considered a relatively homogeneous tumor group from a histopathological perspective, it exhibits marked molecular heterogeneity [4,5]. This heterogeneity is regarded as one of the main determinants of inter-individual differences in the disease course and treatment response.

In this context, receptor tyrosine kinases (RTKs) and their downstream signaling pathways, particularly PI3K/AKT/mTOR and MAPK, play a central role in tumor cell proliferation, invasion, metastasis, and the development of treatment resistance [6]. Therefore, the identification of molecular biomarkers capable of accurately predicting the disease course and survival in HGSC is of critical clinical importance for improving prognostic risk stratification and developing targeted therapeutic strategies [7].

The human epidermal growth factor receptor (HER) family consists of four members, EGFR (HER1), HER2, HER3, and HER4, and mediates a complex signaling network that regulates cellular proliferation, differentiation, and survival [8]. Among these receptors, HER3 (ERBB3) has long been considered catalytically inactive, owing to its weak intrinsic tyrosine kinase activity. However, accumulating evidence indicates that HER3 robustly activates the PI3K/AKT signaling pathway through ligand-dependent (heregulin/NRG1) activation and heterodimerization with HER2 or EGFR [9]. Presence of multiple PI3K-binding motifs within the cytoplasmic domain of HER3 at the center of cellular survival signaling. In HGSC, increased HER3 expression has been associated with enhanced proliferation, strengthened anti-apoptotic signaling, induction of epithelial–mesenchymal transition (EMT), increased metastatic potential, and the development of adaptive resistance to chemotherapy [10,11].

Folate receptor alpha (FOLR1/FRα) is a glycosylphosphatidylinositol-anchored cell surface protein that is highly expressed in serous ovarian carcinomas. By mediating intracellular folate uptake, it supports DNA synthesis and one-carbon metabolic pathways that are essential for tumor cell proliferation [12]. This metabolic advantage confers a proliferative benefit to tumor cells and renders FOLR1 an attractive therapeutic target, particularly for antibody–drug conjugates (ADCs). Nevertheless, the prognostic role of FOLR1 remains controversial, and it is thought to function more as a modulator of oncogenic signaling than as a primary driver oncogene [13].

Emerging evidence suggests that the concomitant high expression of HER3 and FOLR1 within the same tumor cell may represent an aggressive oncogenic phenotype in which metabolic support is integrated with potent proliferative and survival signaling. This interaction provides an important biological model for explaining the invasion, metastasis, and poor prognosis of HGSC.

From a clinical perspective, FIGO stage, residual tumor burden, platinum sensitivity, and BRCA/HRD status are considered principal prognostic determinants of survival in HGSC. However, the observation of substantial survival differences among patients diagnosed at the same stage and treated with similar therapeutic regimens indicates that existing clinicopathological parameters are insufficient to fully capture biological heterogeneity [14]. In this regard, the expression profiles of cell surface molecules such as HER3 and FOLR1 have emerged as candidate biomarkers capable of providing additional prognostic information beyond traditional factors.

Another factor that enhances the clinical relevance of HER3 and FOLR1 is their therapeutic targeting abilities. Mirvetuximab soravtansine, a FOLR1-targeted antibody–drug conjugate, has demonstrated clinical efficacy in phase III trials involving FRα-positive, platinum-resistant HGSC patients and has received FDA approval [15,16]. HER3-targeted agents, including patritumab deruxtecan (HER3-DXd), although not yet approved for ovarian cancer, have shown promising activity against other solid tumors and have highlighted the potential of HER3 expression–based targeted treatment strategies [17,18].

This study aimed to evaluate HER3 and FOLR1 expression in high-grade serous ovarian carcinoma, investigate their associations with clinicopathological characteristics, and determine their prognostic impact on overall survival (OS) and progression-free survival (PFS). In addition, by analyzing the relationship between HER3 and FOLR1, this study sought to explore the biological and clinical significance of their combined assessment. These findings are expected to contribute to improved molecular risk stratification and support the development of HER3- and FOLR1-targeted personalized therapeutic strategies.

## 2. Materials and Methods

### 2.1. Study Design and Data Collection

This retrospective observational study included patients treated and followed up between 1 January 2017 and 1 January 2025 at two tertiary referral centers: Batman Training and Research Hospital and Gazi Yaşargil Training and Research Hospital, Department of Medical Oncology and Pathology. The study cohort consisted of patients with histopathologically confirmed serous ovarian carcinoma, classified according to the World Health Organization (WHO) Classification of Female Genital Tumors, 5th edition [19].

Demographic and clinicopathological data were retrieved from institutional electronic medical records. Collected variables included age, Eastern Cooperative Oncology Group (ECOG) performance status, menopausal status, tumor–node–metastasis (TNM) classification, tumor laterality, tumor size, ovarian surface involvement, use of neoadjuvant chemotherapy (NACT), type of surgery (primary or interval debulking), presence of peritoneal involvement, receipt of adjuvant chemotherapy, and occurrence of disease recurrence or progression during follow-up. Of the 100 patients initially screened, 12 were excluded because of insufficient tumor tissue for immunohistochemical analysis and 22 were excluded because of incomplete clinical follow-up data. The final study population consisted of 66 patients.

Treatment strategies included either primary cytoreductive surgery followed by adjuvant chemotherapy, or platinum- and taxane-based neoadjuvant chemotherapy followed by interval debulking surgery. All patients were monitored regularly using clinical and radiological assessments throughout the treatment and follow-up period. Data regarding postoperative residual tumor burden, platinum sensitivity status, homologous recombination deficiency (HRD) status beyond BRCA mutation, and maintenance therapies (such as PARP inhibitors or bevacizumab) were not consistently available in the medical records and therefore were not included in the analyses.

**Figure 1:**
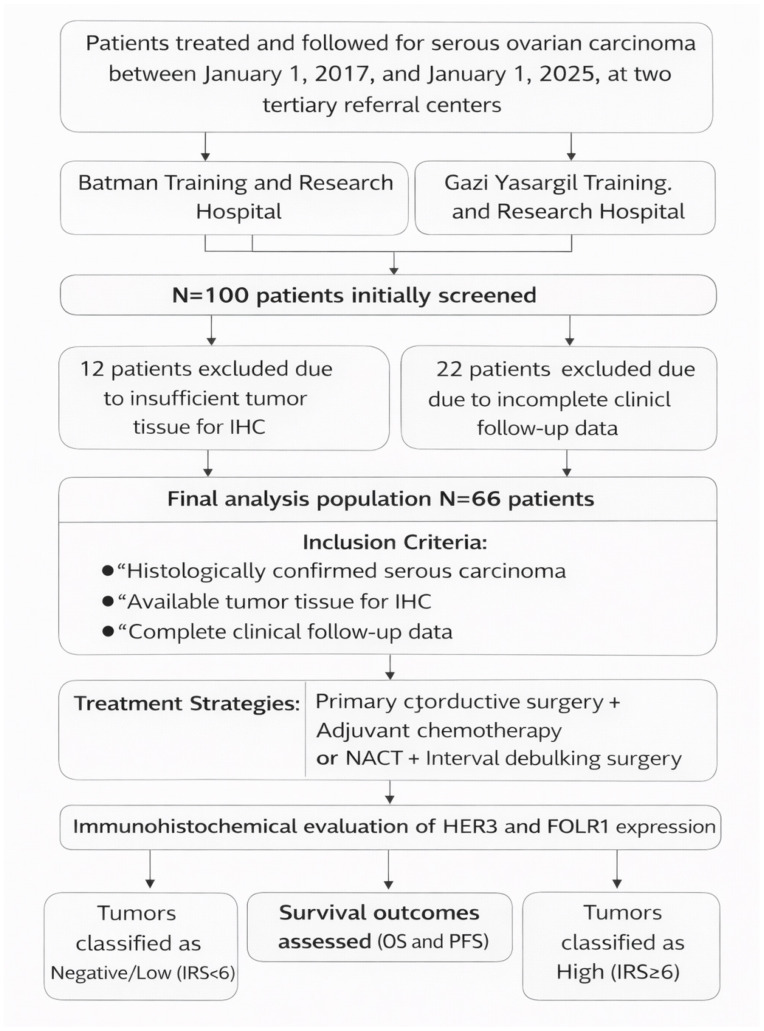
Flow chart illustrating the study design.

Progression-free survival (PFS) was defined as the time from the date of pathological diagnosis to documented disease recurrence, progression, death, or last follow-up, whichever occurred first. Overall survival (OS) was defined as the time from the date of pathological diagnosis to death from any cause or last follow-up (Figure 1).

### 2.2. Immunohistochemical Evaluation of HER3 and FOLR1 Expression

Tumor specimens were re-evaluated using hematoxylin and eosin (H&E) staining, and representative formalin-fixed paraffin-embedded tissue blocks containing viable tumor tissues were selected. Sections of 4–5 μm thickness were prepared and mounted on adhesive-coated slides.

Immunohistochemical staining for HER3 and FOLR1 was performed using validated primary antibodies, according to the manufacturer’s protocol. Tissue sections were incubated with antibodies against HER3 (rabbit monoclonal antibody, clone D22C5, dilution 1:250, Cell Signaling Technology, Danvers, MA, USA) and FOLR1 (mouse monoclonal antibody, clone E8U2F, dilution 1:100, Cell Signaling Technology, Danvers, MA, USA). All staining procedures were performed using a fully automated immunohistochemistry system (Ventana Benchmark Ultra, Roche Diagnostics).

All the stained slides were independently evaluated by four experienced gynecologic pathologists (R.O.Y., S.K., N.D., and A.Y.) who were blinded to the clinical and survival data.

For HER3 immunohistochemistry, membranous staining was considered positive and human breast carcinoma tissue was used as a positive control. For FOLR1 immunohistochemical evaluation, membranous and/or cytoplasmic staining was considered positive, with human placental tissue serving as the positive control.

The staining intensity was scored as 0 (none), 1 (weak), 2 (moderate), or 3 (strong), whereas the proportion of positive tumor cells was scored as 0 (0%), 1 (1–19%), 2 (20–50%), or 3 (>50%). The immunoreactivity score (IRS) was calculated as the product of the staining intensity and proportion score. Tumors were subsequently categorized as negative/low (IRS < 6) or high (IRS ≥ 6) [20].

### 2.3. Statistical Analysis

All statistical analyses were performed using IBM SPSS Statistics for Windows, version 27.0 (IBM Corp., Armonk, NY, USA). The normality of continuous data distribution was assessed using the Kolmogorov–Smirnov or Shapiro–Wilk tests. Continuous variables were summarized as medians with interquartile ranges (IQRs), and categorical variables were expressed as frequencies and percentages.

Comparisons between categorical variables were performed using the chi-square test or Fisher’s exact test. The correlation between HER3 and FOLR1 expression was evaluated using Spearman’s rank correlation coefficient. Survival curves were generated using the Kaplan–Meier method and compared with the log-rank test. Univariate Cox proportional hazards regression analyses were conducted to assess the associations between clinicopathological variables and survival outcomes. Variables showing a *p* value < 0.10 in univariate analyses were subsequently entered into multivariate Cox proportional hazards models to identify independent prognostic factors. The proportional hazards assumption was assessed graphically using log-minus-log survival plots and by time-dependent covariate analysis, and no significant violations were observed. Results are presented as hazard ratios (HRs) with corresponding 95% confidence intervals. A *p* value < 0.05 was considered statistically significant.

## 3. Results

### 3.1. Patient Characteristics

A total of 66 patients with high-grade serous ovarian carcinoma were included in the study. The median age was 58 years (interquartile range [IQR], 31–79 years); 43.9% (n = 29) were younger than 55 years and 56.1% (n = 37) were aged ≥55 years. Most patients were postmenopausal (78.8%, n = 52). Platinum resistance was observed in 34.8% (n = 23) of patients, while 65.2% (n = 43) were platinum-sensitive. Bilateral ovarian involvement was predominant (75.8%, n = 50). Ovarian capsule involvement was most commonly grade 2 (87.9%, n = 58). Peritoneal implants were present in 72.7% of the patients (n = 48). According to the International Federation of Gynecology and Obstetrics staging, 53.0% (n = 35) had stage III disease and 22.7% (n = 15) had stage IV disease. Nodal involvement was detected in 15.2% (n = 10) of the patients, distant metastasis in 21.2% (n = 14), and BRCA positivity in 12.1% (n = 8). Interval debulking surgery was performed in 24.2% (n = 16) of patients, and 40.9% (n = 27) received neoadjuvant chemotherapy.

Based on immunohistochemical evaluation, 62.1% (n = 41) of the patients were classified as HER3-negative/low and 37.9% (n = 25) as HER3-high. A statistically significant association was found between HER3 expression status and the presence of distant metastasis (*p* = 0.025). HER3-high expression was observed more frequently in patients with distant metastasis compared with those without metastasis (36.0% vs. 12.2%). Conversely, HER3-negative/low expression was more common in non-metastatic cases than in metastatic cases (87.8% vs. 64.0%). No significant association was observed with age, menopausal status, tumor laterality, ovarian capsule involvement, implant presence, FIGO stage, nodal involvement, BRCA status, platinum resistance, interval debulking surgery, or neoadjuvant chemotherapy (all *p* > 0.05).

**Figure 2:**
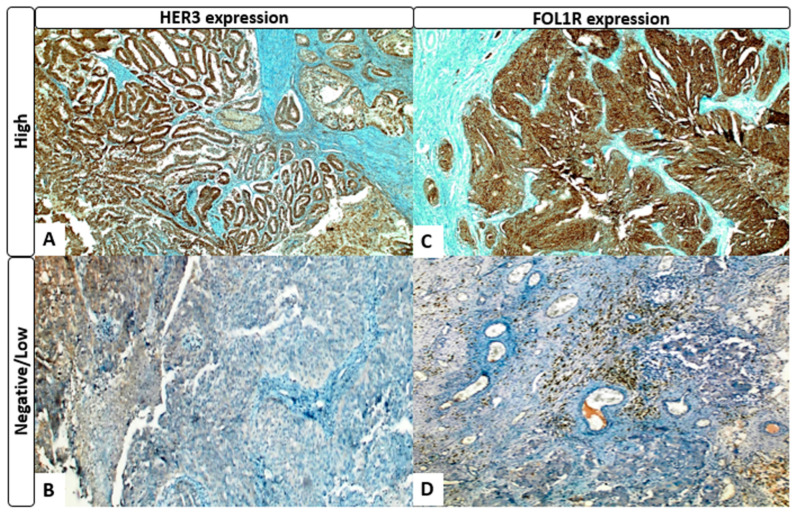
Representative immunohistochemical staining of HER3 and FOLR1 expression in high-grade serous ovarian carcinoma. (**A**) High HER3 expression showing strong diffuse membranous staining in tumor cells. (**B**) Negative HER3 expression with absent or weak staining. (**C**) High FOLR1 expression showing membranous/cytoplasmic staining in tumor cells. (**D**) Negative FOLR1 expression with minimal or no detectable staining (DAB × 200).

For FOLR1, 68.2% (n = 45) were classified as negative/low and 31.8% (n = 21) as high expression. Premenopausal status was significantly more frequent in the FOLR1-high group (38.1% vs. 13.3%; *p* = 0.027). No other clinicopathological variables were significantly associated with FOLR1 expression (all *p* > 0.05) (Table 1, Figure 2).

### 3.2. Survival Analysis

**Figure 3:**
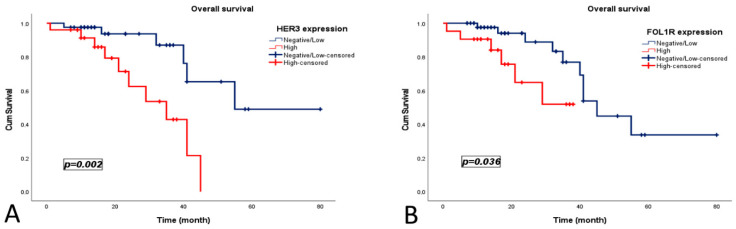
Kaplan–Meier curves showing overall survival according to (**A**) HER3 expression and (**B**) FOLR1 expression in patients with high-grade serous ovarian carcinoma.

During the median follow-up period of 38 months, 16 patients (24.2%) died. The median OS for the entire cohort was 41 months (95% CI, 35.80–46.20). Patients with HER3-high expression had significantly worse OS than those with HER3-negative/low expression (30 vs. 59 months; *p* = 0.002). Similarly, FOLR1-high expression was associated with shorter OS (28 vs. 45 months; *p* = 0.036) (Figure 3).

**Figure 4:**
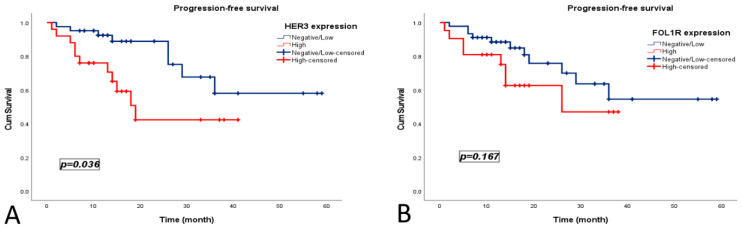
Kaplan–Meier curves showing progression-free survival according to (**A**) HER3 expression and (**B**) FOLR1 expression in patients with high-grade serous ovarian carcinoma.

During follow-up, 47 patients (71.2%) experienced no disease recurrence, whereas 19 patients (28.8%) developed recurrence. The median PFS was 39 months (95% CI, 32.56–46.51). HER3-high expression was associated with significantly shorter PFS (19 vs. 44 months; *p* = 0.036). Although FOLR1-high expression showed a numerically shorter PFS (25 vs. 41 months), this difference was not statistically significant (*p* = 0.167) (Figure 4).

Univariate Cox regression analyses, HER3-high expression was significantly associated with worse OS (HR = 4.684, 95% CI: 1.576–13.926; *p* = 0.005) and PFS (HR = 4.605, 95% CI: 1.557–13.618; *p* = 0.006). FOLR1-high expression was associated with poorer OS (HR = 3.328, 95% CI: 1.009–10.974; *p* = 0.048), but not PFS. The other clinicopathological variables showed no significant association with survival outcomes (Table 2).

In multivariate analyses, HER3 expression remained an independent adverse prognostic factor for both OS (HR = 3.969, 95% CI, 1.273–12.379; *p* = 0.018) and PFS (HR = 4.216, 95% CI: 1.346–13.205; *p* = 0.014). FOLR1 expression did not retain an independent prognostic significance in the adjusted models (Table 3).

## 4. Discussion

In this study, we comprehensively evaluated the prognostic impact of HER3 and FOLR1 expression in high-grade serous ovarian carcinoma (HGSC), in relation to clinicopathological characteristics and survival outcomes. Our findings demonstrate that HER3 expression is a strong and independent adverse prognostic factor for both overall survival (OS) and progression-free survival (PFS). In contrast, although FOLR1 expression was significantly associated with OS, this effect was not observed in multivariate analyses. Moreover, the positive correlation observed between HER3 and FOLR1 expression suggests that these two molecules may act through shared or interacting oncogenic pathways in HGSC.

Although FIGO stage, residual tumor status, platinum sensitivity, and BRCA/HRD status are widely accepted as key prognostic determinants in HGSC, substantial survival heterogeneity persists among patients with similar stage and treatment characteristics [21,22]. This observation highlights the need for more refined molecular biomarkers that can better reflect the intrinsic tumor aggressiveness.

In our cohort, high HER3 expression was significantly associated with distant metastasis and emerged as a strong predictor of poor survival, supporting its role as an indicator of aggressive biological behavior in HGSC.

HER3 (ERBB3), despite its limited intrinsic tyrosine kinase activity, is capable of robust activation of the PI3K/AKT/mTOR signaling pathway through heterodimerisation with HER2 and EGFR. This pathway plays a central role in cellular proliferation, evasion of apoptosis, epithelial–mesenchymal transition, and metastatic progression. Accumulating evidence indicates that HER3 is a critical adaptive node in resistance mechanisms against both cytotoxic chemotherapy and targeted therapies [11,23,24]. Consistent with these data, our finding that high HER3 expression significantly shortened both OS and PFS while retaining independent prognostic significance in multivariable models suggests that HER3 may function not only as a passive biomarker, but also as a functional driver of disease progression in HGSC.

FOLR1 (folate receptor alpha) expression was also associated with OS in our study; however, its impact on PFS did not reach statistical significance. This finding implies that FOLR1 may primarily contribute to tumor biology through metabolic support rather than directly driving aggressive behavior. FOLR1 facilitates intracellular folate uptake, thereby supporting DNA synthesis and one-carbon metabolism; however, its oncogenic influence appears to be more indirect than that of receptors such as HER3, which directly activate major survival signaling pathways [25]. The higher prevalence of FOLR1 expression among premenopausal patients suggests that hormonal or metabolic microenvironmental factors modulate its expression.

The concurrent involvement of proliferative signaling pathways and metabolic adaptation mechanisms in high-grade serous ovarian carcinoma supports the biological relevance of the HER3–FOLR1 interaction. Increased folate uptake mediated by FOLR1 may enhance cellular metabolic capacity, while HER3-driven activation of the PI3K/AKT pathway is known to promote tumor cell survival and proliferation. Collectively, these processes may contribute to a more aggressive tumor phenotype. In the present study, the moderate but statistically significant positive correlation between HER3 and FOLR1 expression reinforces the potential relevance of this interaction and suggests the presence of a coordinated molecular pathway in HGSC.

Antibody–drug conjugates (ADCs) are increasingly being explored as a therapeutic option in gynecologic oncology. HER3-directed ADCs, including patritumab deruxtecan, have shown promising activity across several solid tumors, raising the possibility that HER3 expression may help inform patient selection in future clinical trials [26,27]. Likewise, the demonstrated efficacy of the FOLR1-targeted agent mirvetuximab soravtansine in platinum-resistant high-grade serous carcinoma (HGSC) suggests that FOLR1 may hold potential as a biomarker in prospective treatment stratification strategies.

That said, it is important to clearly distinguish the scope of our findings. The present study assessed the prognostic—not predictive—value of HER3 and FOLR1 expression. We did not evaluate treatment exposure or response to HER3- or FOLR1-targeted therapies in this cohort. Accordingly, any therapeutic implications should be viewed as exploratory and hypothesis-generating rather than practice-changing. Further prospective studies specifically designed to assess predictive relevance in the context of targeted therapies are necessary to determine their true clinical utility.

In addition, several clinically relevant variables, including residual tumor burden, extended homologous recombination deficiency (HRD) status, and maintenance treatment regimens, were not available for analysis. These factors are known to influence survival outcomes and may potentially confound the observed associations. Their absence limits our ability to fully adjust for established prognostic determinants, and therefore the independent prognostic value of HER3 should be interpreted with caution.

This study included the simultaneous assessment of HER3 and FOLR1 expression within the same HGSC cohort, investigation of their biological interplay through correlation analyses, and use of both univariable and multivariable survival models. Additionally, immunohistochemical evaluations were performed independently of the clinical data by experienced pathologists, thereby enhancing the robustness and reliability of the findings.

This study has several limitations. The retrospective nature of the study precludes firm causal conclusions, and the relatively small sample size may have reduced statistical power, especially in subgroup analyses. This limitation may have hindered the detection of more subtle associations involving FOLR1 expression. Accordingly, the lack of a significant relationship between FOLR1 and progression-free survival, as well as the results of correlation analyses, should be interpreted cautiously. The modest number of OS and PFS events results in a limited events-per-variable ratio, which may compromise the robustness and reliability of multivariate Cox regression models and contribute to the wide confidence intervals observed. Although HER3 emerged as an independent prognostic factor in the multivariate Cox models, the number of survival events was modest. The events-per-variable ratio was 8 for OS and 9.5 for PFS, slightly below the conventional 10-event threshold, indicating a potential risk of model instability and overfitting. Therefore, the findings should be interpreted cautiously. The proportional hazards assumption was assessed and no significant violations were identified. Although we aimed to construct parsimonious models, these findings should be considered exploratory and require confirmation in larger, prospective cohorts. Furthermore, the absence of standardized immunohistochemical cut-off values for HER3 and FOLR1 may affect comparability across studies. Nevertheless, the consistency of the survival associations and the biologically plausible correlation between HER3 and FOLR1 support the validity of our results and provide a strong rationale for larger prospective validation studies.

## 5. Conclusions

This study identified HER3 expression as a robust and independent adverse prognostic biomarker in high-grade serous ovarian carcinoma that was significantly associated with both overall and progression-free survival. By contrast, FOLR1 expression had a limited and non-independent prognostic impact, suggesting a predominantly supportive metabolic role. The observed positive correlation between HER3 and FOLR1 indicates a potential integrated biological axis that contributes to tumor aggressiveness. These findings support HER3-based risk stratification and provide a rationale for future prospective validation and targeted therapeutic strategies.

## Figures and Tables

**Table 1:** Association of HER3 and FOLR1 Expression with Clinicopathological Characteristics in Patients with High-Grade Serous Ovarian Carcinoma.

			HER3 Expression			FOLR1 Expression		
		n (%)	Negative/Low n (%)	High n (%)	*p*	Negative/Low n (%)	High n (%)	*p*
**Age**	<55	29 (43.9)	15 (36.6)	14 (56.0)	0.099	20 (44.4)	9 (42.9)	0.559
	≥55	37 (56.1)	26 (63.4)	11 (44.0)		25 (55.6)	12 (57.1)	
**Menopausal status**	Premenopausal	14 (21.2)	8 (19.5)	6 (24.0)	0.446	6 (13.3)	8 (38.1)	0.027
	Postmenopausal	52 (78.8)	33 (80.5)	19 (76.0)		39 (86.7)	13 (61.9)	
**ECOG performance status**	0–1	47 (71.2)	14 (42.4)	8 (32.0)	0.312	18 (40.0)	8 (38.1)	0.455
	≥2	19 (28.8)	19 (47.6)	17 (68.0)		27 (60.0)	13 (61.9)	
**Tumor laterality**	Right	43 (65.2)	3 (7.3)	4 (16.0)	0.625	3 (6.7)	4 (19.0)	0.145
	Left	23 (34.8)	7 (17.1)	2 (8.0)		6 (13.3)	3 (14.3)	
	Bilateral	7 (10.6)	31 (75.6)	19 (76.0)		36 (80.0)	14 (66.7)	
**Ovarian capsule involvement**	1	9 (13.6)	4 (9.8)	2 (8.0)	0.722	5 (11.1)	1 (4.8)	0.330
	2	50 (75.8)	35 (85.4)	23 (92.0)		39 (86.7)	19 (90.5)	
	3	6 (9.1)	2 (4.9)	0 (0.0)		1 (2.2)	1 (4.8)	
**Implant presence**	No	58 (87.9)	12 (29.3)	6 (24.0)	0.432	12 (26.7)	6 (28.6)	0.547
	Yes	2 (3.0)	29 (70.7)	19 (76.0)		33 (73.3)	15 (71.4)	
**Nodal involvement**	No	18 (27.3)	33 (80.5)	23 (92.0)	0.183	36 (80.0)	20 (95.2)	0.103
	Yes	48 (72.7)	8 (19.5)	2 (8.0)		9 (20.0)	1 (4.8)	
**Distant metastasis**	No	5 (7.6)	36 (87.8)	16 (64.0)	0.025	36 (80.0)	16 (76.2)	0.479
	Yes	11 (16.7)	5 (12.2)	9 (36.0)		9 (20.0)	5 (23.8)	
**FIGO stage**	I	35 (53.0)	2 (4.9)	3 (12.0)	0.699	2 (4.4)	3 (14.3)	0.509
	II	15 (22.7)	8 (19.5)	3 (12.0)		8 (17.8)	3 (14.3)	
	III	56 (84.8)	24 (58.5)	11 (44.0)		25 (55.6)	10 (47.6)	
	IV	10 (15.2)	7 (17.1)	8 (32.0)		10 (22.2)	5 (23.8)	
**Interval debulking**	No	52 (78.8)	32 (78.0)	18 (72.0)	0.393	34 (75.6)	16 (76.2)	0.606
	Yes	14 (21.2)	9 (22.0)	7 (28.0)		11 (24.4)	5 (23.8)	
**NACT**	No	58 (87.9)	24 (58.5)	15 (60.0)	0.557	24 (53.3)	15 (71.4)	0.130
	Yes	8 (12.1)	17 (41.5)	10 (40.0)		21 (46.7)	6 (28.6)	
**BRCA status**	Negative	50 (75.8)	34 (82.9)	24 (96.0)	0.114	40 (88.9)	18 (85.7)	0.499
	Positive	16 (24.2)	7 (17.1)	1 (4.0)		5 (11.1)	3 (14.3)	
**Platinum resistance**	No	39 (59.1)	28 (68.3)	15 (60.0)	0.336	29 (64.4)	14 (66.7)	0.544
	Yes	27 (40.9)	13 (31.7)	10 (40.0)		16 (35.6)	7 (33.3)	

**Abbreviations**: ECOG, Eastern Cooperative Oncology Group; FIGO, International Federation of Gynecology and Obstetrics; FOLR1, folate receptor alpha; NACT, neoadjuvant chemotherapy; BRCA, breast cancer susceptibility gene. Correlation analysis revealed a statistically significant positive association between HER3 and FOLR1 expression (r = 0.338; *p* = 0.005), indicating that increased HER3 expression was associated with a concomitant increase in FOLR1 expression.

**Table 2:** Univariate Cox Regression Analysis for Overall Survival and Progression-Free Survival.

	Univariate for OS			Univariate for PFS		
	HR	95% CI	*p*	HR	95% CI	*p*
**Age**	1.29	0.443–3.758	0.640	1.159	0.389–3.451	0.791
**Menopausal status**	1.569	0.351–7.017	0.556	1.192	0.260–5.471	0.821
**ECOG performance status**	1.33	0.12–4.21	0.324	0.88	0.188–2.145	0.654
**Platinum resistance**	0.892	0.307–2.590	0.834	0.767	0.262–2.245	0.629
**Tumor localization**	0.927	0.489–1.757	0.816	0.904	0.468–1.748	0.765
**Ovarian capsule involvement**	2.599	0.566–11.925	0.219	2.406	0.636–9.105	0.196
**Implant presence**	1.055	0.363–3.061	0.922	1.232	0.409–3.707	0.711
**FIGO stage**	1.018	0.504–2.057	0.960	1.346	0.655–2.763	0.419
**Nodal involvement**	0.290	0.038–2.212	0.232	0.422	0.055–3.261	0.408
**Distant metastasis**	1.293	0.465–3.601	0.622	2.119	0.750–5.987	0.156
**BRCA status**	0.363	0.047–2.799	0.331	0.541	0.070–4.168	0.556
**Interval debulking surgery**	0.463	0.131–1.643	0.234	0.405	0.109–1.501	0.176
**Neoadjuvant chemotherapy**	0.393	0.134–1.151	0.089	0.432	0.147–1.268	0.127
**HER3 expression**	4.684	1.576–13.926	0.005	4.605	1.557–13.618	0.006
**FOLR1 expression**	3.328	1.009–10.974	0.048	2.266	0.758–6.774	0.143

**Abbreviations**: OS, overall survival; PFS, progression-free survival; HR, hazard ratio; CI, confidence interval; ECOG, Eastern Cooperative Oncology Group; FIGO, International Federation of Gynecology and Obstetrics; HER3 (ERBB3), human epidermal growth factor receptor 3; FOLR1, folate receptor alpha; BRCA, breast cancer susceptibility gene.

**Table 3:** Multivariate Cox Regression Analysis for Overall Survival and Progression-Free Survival.

	Multivariate for OS			Multivariate for PFS		
	HR	95% CI	*p*	HR	95% CI	*p*
**FOLR1 expression**	2.135	0.621–7.343	0.229	1.349	0.427–4.255	0.610
**HER3 expression**	3.969	1.273–12.379	0.018	4.216	1.346–13.205	0.014

**Abbreviations**: OS: overall survival; PFS: progression-free survival; HR: hazard ratio; CI: confidence interval; HER3 (ERBB3): human epidermal growth factor receptor 3; FOLR1: folate receptor alpha.

## Data Availability

The data presented in this study are available from the corresponding author upon reasonable request and with permission from the relevant institution.

## Abbreviations

ADC	antibody–drug conjugate
AKT	protein kinase B
BRCA	breast cancer susceptibility gene
CI	confidence interval
ECOG	Eastern Cooperative Oncology Group
EGFR	epidermal growth factor receptor
EMT	epithelial–mesenchymal transition
FIGO	International Federation of Gynecology and Obstetrics
FOLR1 (FRα)	folate receptor alpha
HER	human epidermal growth factor receptor
HER3 (ERBB3)	human epidermal growth factor receptor 3
HGSC	high-grade serous ovarian carcinoma
HR	hazard ratio
HRD	homologous recombination deficiency
IHC	immunohistochemistry
IQR	interquartile range
IRS	immunoreactivity score
MAPK	mitogen-activated protein kinase
mTOR	mammalian target of rapamycin
NACT	neoadjuvant chemotherapy
OS	overall survival
PFS	progression-free survival
PI3K	phosphoinositide 3-kinase
RTK	receptor tyrosine kinase
TNM	tumor–node–metastasis
WHO	World Health Organization
